# A taxonomy of Chinese hospitals and application to medical dispute resolutions

**DOI:** 10.1038/s41598-022-23147-3

**Published:** 2022-10-29

**Authors:** Mengxiao Wang, Hanqing Zhao, Chengxiang Tang, Yu Sun, Gordon G. Liu

**Affiliations:** 1 School of Public Administration, Southwestern University of Finance and Economics, 555 Liutai Street, Chengdu, 611130 China; 2 Institute of Health Policy and Hospital Management, Sichuan Academy of Medical Sciences & Sichuan Provincial People’s Hospital, Chengdu, China; 3 Centre for the Health Economy, Business School & Australian Institute of Health Innovation, Macquarie University, Sydney, Australia; 4 China Center for Health Economic Research, National School of Development, Peking University, 5 Yiheyuan Road, Beijing, 100871 China

**Keywords:** Health policy, Health care economics

## Abstract

Medical disputes can be viewed as a negative indicator of health care quality and patient satisfaction. However, dispute prevention from the perspective of systematic supervision is unexplored. This study examines hospital clustering based on diagnosis-related group (DRG) indicators and explores the association between hospital clusters and medical disputes. Health administrative data from Sichuan Province in 2017 were used. A twostep cluster analysis was performed to cluster hospitals based on DRG indicators. A multiple regression analysis was conducted to evaluate the relationship between clusters and the incidence/number of medical disputes. The 1660 hospitals were grouped into three DRG clusters: basic (62.5%, n = 1038), diverse (31.0%, n = 515), and lengthy (6.4%, n = 107). After adjusting for covariates, the diverse hospitals were associated with an increased probability of having medical disputes (OR 5.24, 95% CI 2.97–9.26), while the diverse and lengthy hospitals were associated with a greater number of medical disputes (IRR 10.67, 95% CI 6.58–17.32; IRR 4.06, 95% CI 1.22–13.54). Our findings highlighted that the cluster-level performance of hospitals can be monitored. Future studies could examine this relationship using a longitudinal design and explore ways to reduce medical disputes in hospitals.

## Introduction

Concerns regarding the unsustainable growth of health care expenditures make cost containment one of the major targets of health care reform worldwide. Multiple policies have been adopted in different countries, including payment reform, managed care, and cost sharing. However, empirical evidence shows that only some of these policies are effective and that their effects are context-dependent^1^. Health professionals have expressed concerns that these policies can negatively affect healthcare quality, reduce patient satisfaction by decreasing patients’ medical resources, and deteriorate the relationships between physicians and patients^2^.

The overall number of medical disputes has been increasing worldwide^3^, which has raised concerns about healthcare quality and safety^4^. Inadequacy in quality of health services is the major cause of medical litigations^5^. Multiple dispute resolution approaches have been proposed in China, including negotiation, people’s mediation, administrative mediation, and litigation^6^. The four dispute resolution approaches are given different priorities: when medical dispute occurs, hospitals and patients can choose from negotiation, people’s mediation, and administrative mediation first, while negotiation is only applicable for small amount of compensation with a cap line; if the aforementioned three resolutions fail, either the hospital or patient can file a lawsuit. Hospitals are required to report their dispute information to health authorities according to medical quality and safety incident reporting regulations^7, 8^.

Diagnosis-related groups (DRGs) were first developed to describe and measure health care services, and they later became a part of the payment systems of many countries. The DRG classification system can be used to adjust patient risks through case mixing and improve the consistency of hospital performance, thus facilitating health planning and management activities^9, 10^. To increase transparency, efficiency, and quality of care, the DRG payment method has been widely implemented as a means to monitor and control the utilization of health services in both developed and developing countries^11, 12^. Evaluations of DRG systems have been conducted, focusing on medical cost, length of stay, readmissions and so on^13–15^. The effects of DRG-based systems on healthcare quality and patient outcomes are quite mixed and far from conclusive^16^.

Studies have shown that healthcare providers may change their behaviours in reaction to cost containment policies^17^. Intentional upcoding and overtreatment are quite common in France and Germany^18^. In China, physicians sometimes choose to accept fewer local patients covered by public health insurance, reject seriously ill patients, and prescribe expensive drugs and procedures^2^. Considering that cost containment policies influence the intensity and scope of the services offered by healthcare providers, it is argued that cost-containment measures may interact with tort systems and that third-party payors should bear responsibility in case medical harm occurs due to cost and utilization controls^19^. Despite the argument on whether ex post liability should be assigned to health providers or insurers, research on capacity building and ex ante supervision at the hospital level to reduce medical malpractice based on DRG systems is quite limited.

DRG systems have been widely adopted in many countries, but their practical application to the field of medical dispute prevention requires certain prerequisites. As pointed out in earlier studies, DRG-based inpatient service management requires a good information system, high-quality electronic medical records, and a management team^9^. In 2011, the Ministry of Health (later known as the National Health Commission) advocated for the implementation of DRGs in health systems across China to facilitate evaluations of the quality and performance of hospitals as well as reform payment mechanisms^20^. Currently, DRG evaluation is an important part of the overall assessment of China’s healthcare capacity and quality^21^. Thus, our study focuses on DRG-based hospital supervision and medical dispute prevention.

Taxonomic analyses can be used to classify organizations into different groups, and the results can provide a framework to assess performance. Through clustering analysis methods, cluster solutions are generated and health institutions are characterized into distinct groups accordingly. With the ongoing efforts to integrate health care services (bring together hospitals, physicians, etc.), the performance of accountable care organizations (ACOs) has recently received much attention. Based on resource dependence theory and institutional theory, ACOs can be classified into three different clusters according to eight characteristics (size, scope of services offered, and so on) through a two-step cluster analysis^22^. Using hierarchical clustering methods, hospitals that participate in ACOs can be classified into five distinct groups^23^.

Hospital accreditation has been widely used as a tool to improve quality and safety of health care^24, 25^. To facilitate the macro management of medical and healthcare services, hospitals in China are divided into three broad categories based on their functions and tasks through accreditation^26^. However, this classification may not be suitable in the context of medical dispute prevention, as tort law requires a certain quality and standard of health care regardless of a hospital’s administrative classification or costs. Given that the Chinese government started evaluating the performance of public hospitals at the national level in 2019^27^, few studies have explored clusters of Chinese hospitals using the taxonomy method. As DRG indicators reflect the characteristics of medical services from multiple perspectives, additional analyses can be conducted to classify hospitals into different groups based on DRG attributes.

This study aims to make three contributions: (i) we attempt group hospitals in Sichuan Province, China based on DRG indicators, and we describe the characteristics of different clusters; (ii) we explore the associations between hospital clusters and the incidence of medical disputes; and (iii) we examine the degree to which belonging to a particular hospital cluster is associated with a hospital’s number of medical disputes.

## Methods

### Study design

Our study focused on Sichuan Province, which is located in Southwest China; as of 2019, the province had a population of 83.75 million, and the number of hospitals in the province reached 2435 in 2020^28^. Sichuan Province was among the first provinces to apply DRGs to the context of health administration in China and completed a DRGs application platform in early 2017. This study employed three administrative datasets aggregated at the hospital level from Sichuan Province, using data from 2017. The first dataset covered DRGs information, which was derived from the front page of the medical records of the discharged cases of each hospital. The second dataset was based on the Medical and Healthcare Institutions Statistics Monthly Report, and it documented the total number of medical disputes in hospitals and different medical dispute resolutions. The third dataset was collected from the Medical and Healthcare Institutions Statistics Annual Report, and it contained basic hospital characteristics. After these three datasets were combined, a total of 1660 hospitals were included in our analysis. Moreover, we included county-level data from the statistical yearbook of Sichuan Province to control the features of the counties where the examined hospitals were located, including per capita GDP, urbanization rate, and population.

The access to relevant data was granted by Health Information Center of Sichuan Province under the research agreement between Peking University and Health Information Center of Sichuan Province. The data were anonymized and no patient was involved.

### Variables and measurements

#### Medical dispute resolutions

Our data contained the total number of medical disputes in each hospital and the number of disputes settled through different approaches, including malpractice litigation, third-party mediation (usually via a people’s mediation committee), administrative mediation, and negotiation. As disputes information in each hospital was recorded on a monthly basis, we used the sum of the number of disputes over twelve months as the annual number of disputes. Two sets of dispute resolution variables were used as dependent variables in our study. The first set of variables captured the incidence of medical disputes; this variable was equal to 1 if there was a dispute and 0 otherwise. The second set of variables measured the actual number of medical disputes overall and those settled through each approach. We used listwise deletion to address missing data, and the working sample for our dispute analysis contained 1023 hospitals.

#### DRG indicators

Our DRG indicators were calculated according to the Diagnosis Related Groups Sichuan Version (SC-DRGs), which was developed based on the Medicare Severity-Diagnosis Related Groups of the United States and case studies from Beijing and other cities in China. The DRG grouping model contained one advanced grouping, 25 major diagnosis categories, and 753 DRG groups. Additional details on DRG groupings have been published elsewhere^29^. Five indicators were constructed: DRG volume, the case-mix index (CMI), the cost efficiency index (CEI), the time efficiency index (TEI), and the mortality rate of low-risk cases (MLR). The detailed calculation methods employed followed those of a prior study^30^.

After a risk adjustment based on the DRG classification system, the five indicators were used to evaluate the performance of the examined hospitals^21^. A larger DRG volume indicated a wider treatment scope. A high CMI score indicated that the patients being treated in a hospital were more severely ill and consumed more medical resources than those being treated in other hospitals. Higher CEIs and TEIs indicated low levels of cost and time efficiency, respectively. The MLR was used to show hospital safety^30^.

#### Hospital characteristics

We also controlled for hospital information, including the type, ownership, and number of operating years of each hospital as well as the number of physicians and beds. The hospital type variable was made up of three categories (general, traditional Chinese medicine, and specialty) and coded as a set of dummy variables; each was equal to 1 to indicate the corresponding category and 0 otherwise. Hospital ownership was classified as public, private nonprofit, and private for-profit and coded as a set of binary variables. The number of beds in each hospital was used as a proxy for hospital size, while the number of physicians reflected the hospital’s labour input. As established facilities were found to have better management practices^31^, each hospital’s number of operating years since its establishment was also included as a confounding variable.

### Data analysis

#### Cluster analysis

Cluster analysis was performed using SPSS (Version 26.0). Clusters of DRG measures were identified using the SPSS two-step clustering algorithm. This algorithm is designed to handle large datasets and can help determine the optimal number of clusters without a predetermined number^32^. In the first step of this process, the cases were sorted into preclusters; then, they were clustered using hierarchical clustering in the second step. The variables to be clustered were standardized to remove scale effects. The Bayesian information criterion (BIC) was used to determine the best solution; a smaller BIC indicates a better model fit. Since cluster solutions can be influenced by the order of data, the cluster analysis was run four times^33^. The first run was conducted using the original data order, and the latter three runs were conducted with the data in random orders.

To assess the internal validation of the cluster solution, a discriminant analysis was conducted^23^. We performed statistical analyses to investigate the relationships between the examined hospital characteristics and cluster outcomes using chi-square tests and ANOVAs for categorical and continuous variables, respectively.

#### Regression analysis

A regression analysis was conducted using STATA (version 15). Hospital groups based on the cluster solution were coded as binary variables and included as independent variables in the regression models. The impacts of different hospital clusters on the incidence of medical disputes were analysed using a logistic regression model while controlling for hospital characteristics. Odds ratios (ORs) were reported in the main text and average marginal effect results were included in the supplementary material. Given that the variance in the number of disputes was more than twice the mean, we employed a negative binomial model to analyse the relationship between the number of disputes in different hospital clusters and reported incidence rate ratios (IRRs) of the models. Due to the great heterogeneity in socioeconomic characteristics across Sichuan, the standard errors of the regression models were clustered by county.

## Results

### Taxonomy analysis of hospitals in China

The sample hospitals varied greatly in terms of indicators constructed based on SC-DRGs system (see Figs. S1, S2). Four cluster analyses produced three clusters with stable centroids. The first three cluster solutions were used in later analyses. A discriminant analysis of the three-cluster solution showed a 95.7% rate of correct classification, indicating that this solution was internally valid.

Table 1 presents the mean values of the clustering variables and the statistical tests across the clusters. An ANOVA was performed, and the results suggested that significant differences existed across the clusters (p < 0.01). Among all 1660 hospitals, three clusters were identified: basic (with moderate DRG volume), diverse (with large DRG volume), and lengthy (with low time efficiency). The basic cluster comprised the largest proportion of hospitals (1038, 62.5%) and had a moderate DRG volume. Moreover, the basic cluster had the lowest CMI and CEI, indicating that on average, the patients treated in these hospitals were not that severely ill and that the cost efficiency of the hospitals was high. The diverse cluster (515, 31.0%) had the greatest DRG volume, with an average of 344.34. The lengthy cluster (107, 6.4%) featured a high TEI, which indicated that the hospitals in this group were much less time efficient than the others. Given that the majority of the hospitals had zero mortality cases in low-risk groups, the high MLR of the lengthy group was driven by several hospitals with high MLRs, which deserves particular attention from the perspective of patient safety.

**Table 1:** Hospital clusters derived from cluster analysis.

Characteristics	Basic	Diverse	Lengthy
n	1038	515	107
%	62.5	31	6.4
DRG volume	**72.18**	**344.34**	**29.11**
Cost efficiency index	**0.48**	**0.69**	**1.16**
Time efficiency index	**0.95**	**1.02**	**2.51**
Case-mix index	**0.60**	**0.82**	**0.74**
Mortality rate of low-risk groups (%)	0.01	0.02	**0.26**

An ANOVA was used to test for overall differences across all the clusters, and a Tukey’s honestly significant difference test was used to examine significant differences in means between individual clusters.

The bold values are significantly different from those of other clusters at the p < 0.01 level.

### Hospital characteristics and dispute information according to clusters

Table 2 presents the hospital characteristics across the three clusters. Significant differences were found in all respects across the hospital clusters (*p* < 0.01). The basic and diverse clusters were both dominated by general hospitals, which accounted for 73.9% and 63.5% of these groups, respectively, while the lengthy cluster was dominated by specialty hospitals, which accounted for 72.0% of this group. The hospitals in the diverse cluster were mostly public (68.5%) and large in terms of the number of hospital beds (457 on average) and number of physicians (132 on average). The diverse group was also made up of hospitals that were longer-established than their counterparts. The basic and lengthy clusters were mainly made up of private hospitals. The hospitals in the basic cluster were relatively small, with 72 beds and 13 physicians on average. The lengthy cluster ranked in the middle regarding the number of beds, number of physicians, and operating years, and the hospitals in this cluster were mostly located in areas with high per capita GDP, high urbanization rates, and large populations. Overall, there were 2572 medical disputes in our sample. A large proportion of the hospitals had no disputes in 2017, while some hospitals had more than 40 disputes in one year (see Fig. S3). Compared with the other two groups, hospitals in the diverse group had the largest number of medical disputes. Across the three clusters, the negotiation approach accounted for the largest proportion (over 60%).

**Table 2:** Hospital characteristics and dispute information according to hospital clusters.

Variables	Basic	Diverse	Lengthy
**Hospital characteristics**			
Type of medical facility, n (%)			
General hospitals	767 (73.9)	327 (63.5)	30 (28.0)
Traditional Chinese medicine hospitals	64 (6.2)	105 (20.4)	0 (0.0)
Specialty hospitals	207 (19.9)	83 (16.1)	77 (72.0)
Ownership, n (%)			
Public	175 (16.9)	353 (68.5)	45 (42.1)
Private nonprofit	275 (26.5)	62 (12.0)	23 (21.5)
Private for-profit	588 (56.6)	100 (19.4)	39 (36.4)
Other features, mean (sd)			
Beds	72 (48)	457 (498)	260 (301)
Physicians	13 (12)	132 (189)	22 (29)
Operating years	16 (18)	40 (27)	22 (21)
Per capita GDP (in 1000 RMB)	50 (27)	54 (30)	61 (32)
Urbanization rate (%)	54.6 (20.7)	58.5 (22.6)	64.1 (25.5)
Population (in 1000 s)	658 (361)	723 (384)	833 (412)
Dispute information, n (%)			
Total disputes	66	2469	37
Medical malpractice	5 (7.6)	224 (9.1)	1 (2.7)
Third-party mediation	10 (15.2)	515 (20.9)	2 (5.4)
Administrative mediation	10 (15.2)	208 (8.4)	7 (18.9)
Negotiation	41 (62.1)	1522 (61.6)	27 (73.0)

Chi-square tests and ANOVAs were used to test for significant differences in the categorical and continuous variables, respectively, across the clusters.

### Association between hospital clusters and incidence of medical disputes

Table 3 reports the associations between hospital clusters and the probability of medical disputes. The results showed that the hospitals in the diverse cluster were more likely to have medical disputes than those in the basic cluster (OR 5.24, 95% CI 2.97–9.26), the probability increased by 14.6 percentage points on average (see Table S1 for average marginal effect outcomes). No significant differences in the likelihood of medical disputes were found between the hospitals in the lengthy and basic clusters. Regarding the different dispute resolution approaches, the hospitals in the diverse cluster were significantly more likely to use all four approaches than the hospitals in the basic cluster and the probability of negotiation increased the most (16.3 percentage points). The hospitals in the lengthy group were more likely to use administrative mediation to solve medical disputes (OR 7.10, 95% CI 2.40–21.01) than those in the basic cluster, the probability increased by 12.6 percentage points on average; however, no significant difference was found regarding the other approaches. Overall, hospitals with more physicians (OR 1.01, 95% CI 1.01–1.02) and longer operating years (OR 1.02, 95% CI 1.01–1.03) were more likely to have medical disputes. Being private was associated with lower probability to use third-party mediation. Hospitals with larger number of beds were more likely to use third-party mediation.

**Table 3:** Association between hospital clusters and the incidence of medical disputes.

	(1)	(2)	(3)	(4)	(5)
	Total disputes	Medical malpractice	Third-party mediation	Administrative mediation	Negotiation
Mean	0.26	0.08	0.12	0.09	0.21
SD	0.44	0.28	0.32	0.28	0.41
Diverse	5.24^#^ (2.97, 9.26)	10.31^#^ (3.34, 31.83)	7.60^#^ (3.31, 17.44)	7.53^#^ (3.63, 15.60)	6.88^#^ (3.69, 12.83)
Lengthy	2.74 (0.84, 8.97)	2.39 (0.21, 26.78)	1.02 (0.18, 5.80)	7.10^#^ (2.40, 21.01)	2.63 (0.59, 11.74)
Private non-profit	0.55 (0.25, 1.22)	0.21 (0.02, 1.90)	0.12^+^ (0.03, 0.44)	0.41 (0.12, 1.45)	0.56 (0.24, 1.31)
Private for-profit	0.77 (0.41, 1.44)	1.18 (0.37, 3.79)	0.18^+^ (0.06, 0.54)	0.83 (0.34, 2.07)	0.70 (0.35, 1.41)
Beds	1.00 (0.89, 1.13)	0.97 (0.84, 1.12)	1.22^#^ (1.10, 1.35)	0.94 (0.84, 1.06)	0.98 (0.87, 1.12)
Physicians	1.01^#^ (1.01, 1.02)	1.00 (1.00, 1.01)	1.00 (0.99, 1.00)	1.01^#^ (1.00, 1.01)	1.01^+^ (1.00, 1.01)
Operating years	1.02^+^ (1.01, 1.03)	1.02^+^ (1.01, 1.04)	1.00 (0.99, 1.02)	1.01* (1.00, 1.03)	1.02^#^ (1.01, 1.03)

Odds ratios (ORs) are reported; 95% confidence intervals (CIs) are in parentheses. All the regressions controlled for GDP per capita, urbanization rate, and population as well as a traditional Chinese medicine hospital dummy and a specialty hospital dummy. Number of beds were measured in units of 100 in the models. The standard errors were clustered at the county level.

*p < 0.05, ^+^*p* < 0.01, and ^#^*p* < 0.001.

### Association between hospital clusters and number of medical disputes

Table 4 demonstrates the associations between hospital clusters and the total number of medical disputes/number of medical disputes settled through the different approaches. Each hospital had an average of 2.5 medical disputes. Compared with the hospitals in the basic cluster, the hospitals in the diverse and lengthy clusters exhibited numbers of medical disputes that were increased by a factor of 10.67 (IRR = 10.67, 95% CI 6.58–17.32) and 4.06 (IRR = 4.06, 95% CI 1.22–13.54), respectively. For hospitals in the diverse group, the results regarding the different resolution approaches showed a pattern similar to that of results of the incidence analysis. The lengthy group demonstrated larger number of medical disputes in terms of administrative mediation (IRR = 14.43, 95% CI 2.90–71.80) and negotiation (IRR = 4.25, 95% CI 1.12–16.06), while no significant results were found for the other two approaches. Hospitals with more physicians and longer operating years showed larger number of medical disputes. Compared with public hospitals, private non-profit hospitals had fewer medical disputes regarding third-party mediation. Hospitals with larger number of beds had more medical disputes solved using third-party mediation.

**Table 4:** Association between hospital clusters and number of medical disputes.

	(1)	(2)	(3)	(4)	(5)
	Total disputes	Medical malpractice	Third-party mediation	Administrative mediation	Negotiation
Mean	2.51	0.22	0.52	0.22	1.55
SD	7.68	1.43	2.14	0.96	5.76
Diverse	10.67^#^ (6.58, 17.32)	15.53^#^ (4.93, 48.90)	11.81^#^ (5.26, 26.51)	8.16^#^ (3.50, 19.03)	9.62^#^ (5.35, 17.32)
Lengthy	4.06* (1.22, 13.54)	2.63 (0.24, 29.38)	0.46 (0.07, 3.00)	14.43^+^ (2.90, 71.80)	4.25* (1.12, 16.06)
Private non-profit	0.68 (0.33, 1.43)	0.16 (0.02, 1.31)	0.22* (0.06, 0.80)	0.46 (0.10, 2.02)	0.78 (0.32, 1.88)
Private for-profit	0.67 (0.37, 1.23)	1.54 (0.53, 4.44)	0.34 (0.12, 1.00)	1.27 (0.45, 3.59)	0.57 (0.26, 1.22)
Beds	1.01 (0.93, 1.09)	1.00 (0.89, 1.14)	1.18* (1.03, 1.34)	0.95 (0.85, 1.05)	1.00 (0.92, 1.09)
Physicians	1.01^#^ (1.00, 1.01)	1.01* (1.00, 1.01)	1.00 (1.00, 1.00)	1.01^#^ (1.00, 1.01)	1.01^#^ (1.00, 1.01)
Operating years	1.02^#^ (1.01, 1.03)	1.01* (1.00, 1.03)	1.01^+^ (1.00, 1.02)	1.01 (1.00, 1.03)	1.02^#^ (1.01, 1.03)

Incidence rate ratios (IRRs) are reported; 95% confidence intervals (CIs) are in parentheses. All the regressions controlled for GDP per capita, urbanization rate, and population as well as a traditional Chinese medicine hospital dummy and a specialty hospital dummy. Number of beds were measured in units of 100 in the models. The standard errors were clustered at the county level.

*p < 0.05, ^+^*p* < 0.01, and ^#^*p* < 0.001.

## Discussion

To the best of our knowledge, this study is among the first to examine the relationship between hospital clusters and medical disputes in China. Using data from Sichuan Province, China, we identified three clusters based on DRG indicators: basic, diverse, and lengthy. The basic cluster (62.5%) accounted for the largest percentage of the studied hospitals, and this was followed by the diverse cluster (31.0%) and the lengthy cluster (6.4%). We also found that the hospitals in the diverse cluster were more likely to be involved in medical disputes, and the hospitals in the diverse and lengthy clusters had more disputes than those in the basic cluster.

Comparing with studies using taxonomy method to analyse medical institutions in the United States^22, 23^, our study extended the application of this method with DRG indicators as main variables and tested the association of cluster outcomes with medical disputes in China. As DRGs classification systems generally increase the consistency of hospitals and DRG indicators can reflect hospitals’ capacity and performance, our cluster outcomes can provide a relatively robust base for future analysis. With systematic interventions recognized as an important approach to reduce medical errors^34^, the strong association between the cluster outcome and number of medical disputes indicate that targeted interventions can be carried out within specific clusters to reduce the number of medical disputes. Earlier studies demonstrate that hospital management can play a role in dispute prevention^35^; our study shows that hospital taxonomy can be used to provide a policy framework and contribute to dispute prevention from healthcare system level.

In contrast to the existing hospital classification and management system of China, our DRGs-based hospital taxonomy could contribute to implementing a real-time dynamic management approach based on objective hospital performance data. The classification of hospitals in China remained the same until the second wave of hospital accreditation, which occurred in 2011 and allowed secondary hospitals to become tertiary hospitals if they matched the relevant criteria^36, 37^. As pointed out in previous research, hospitals may lose momentum to change after accreditation and the effect of accreditation can fade over time^24^. With the increasing availability of data, our taxonomy approach can help establish a more responsive management system that does not use infrequently updated accreditation information. Thus, health administrative departments could play a more proactive role in selecting major supervision subjects and aligning incentives or regulations accordingly.

Our study contributed to the awareness of prevalence of medical disputes in China and highlighted the importance of prevention ex ante. Previous studies have focused on medical malpractice litigation and people’s mediation^5, 38^; however, we found that most disputes are solved through negotiation and third-party mediation. We confirm that third-party mediation helps ease the burden on health administrative departments and legal systems. Moreover, the high proportion of negotiation cases highlighted that the actual number of disputes solved at the hospital level could be much greater than that revealed by the literature based on retrospective analyses of medical disputes settled through other approaches. Our findings suggested that more attention should be given to medical dispute prevention and resolution mechanisms at the hospital level. For hospital managers, developing monitoring indicators regarding dispute and compensation amount to improve hospital performance is worth considering.

This study is relevant for researchers, healthcare providers, and policy makers. First, we identified the relationships between DRGs-based hospital clusters and the incidence and number of medical disputes. Future studies using DRGs information could further explore the relationship between hospital clusters and patient safety/quality measures. Second, we found that hospitals in diverse and lengthy clusters are associated with a larger number of medical disputes. More policy intervention plans could be implemented for these hospitals to reduce medical disputes and improve their quality and safety. Considering the prerequisites of the application of DRGs-based management, it is also important for policymakers to pay more attention to establishment of digital management platform to lay a foundation for targeted intervention. Third, apart from analysis on disputes within one hospital, it is also important for hospital managers/medical staff to report their quality and safety information to administrative institutions^39^, which can help generate prevention mechanisms at system level and within hospitals. More effort is needed to improve the incentives and procedures of dispute reporting.

Our study has several limitations. First, this study was based on Sichuan data only; although these data are representative of China in terms of demographic and economic characteristics^40^, we should be cautious in generalizing these results to hospitals in other regions of China. As 2017 was the first year of DRGs implementation in Sichuan, our study sample may be comprised of hospitals with relatively good information systems and management; thus, the number of disputes could be biased downward. Second, we evaluated the associations between DRGs-based hospital clusters and the incidence and number of medical disputes with only cross-sectional data. Future research should examine this relationship using longitudinal datasets.

## Conclusion

Our study contributes to the understanding of the use of DRGs evaluations to strengthen supervision of hospitals and enhance medical disputes prevention at system level. Hospitals classified as diverse and lengthy have more medical disputes than those classified as basic. Future analyses could examine this relationship using a longitudinal design and explore interventions for target groups.

## Supplementary Information

Supplementary Information.

## Data Availability

The data that support the findings of this study are available from Health Information Center of Sichuan Province but restrictions apply to the availability of these data, which were used under license for the current study, and so are not publicly available. Data are however available from the corresponding authors upon reasonable request and with permission of the Health Information Center of Sichuan Province.
