# Supplementary material for: A taxonomy of Chinese hospitals and application to medical dispute resolutions
Source: Sci Rep. 2022 Oct 29;12:18234. doi: 10.1038/s41598-022-23147-3 (PMC9617920; doi:10.1038/s41598-022-23147-3)
Supplement: Supplementary file 1 — Supplementary Information. [file 41598_2022_23147_MOESM1_ESM.docx]

**Supplementary materials**

Fig. S1. CMI and DRG volume of the 1,660 hospitals in Sichuan Province in China, 2017

Fig. S2. Time efficiency and cost efficiency of the 1,660 hospitals in Sichuan Province in China, 2017

**Notes** TEI = time efficiency index; CEI = cost efficiency index.

Fig. S3. Frequency distribution of the total number of disputes of the 1,023 hospitals in Sichuan Province in China, 2017

**Tab. S1** Association Between Hospital Clusters and the Incidence of Medical Disputes

|  | (1) | (2) | (3) | (4) | (5) |
| --- | --- | --- | --- | --- | --- |
|  | Total disputes | Medical malpractice | Third-party mediation | Administrative mediation | Negotiation |
| Diverse | 0.146^#^ | 0.132^#^ | 0.140^#^ | 0.130^#^ | 0.163^#^ |
|  | (0.088,0.204) | (0.068,0.197) | (0.084,0.197) | (0.085,0.175) | (0.102,0.225) |
| Lengthy | 0.089 | 0.049 | 0.001 | 0.126^#^ | 0.082 |
|  | (-0.019,0.197) | (-0.088,0.187) | (-0.119,0.122) | (0.059,0.193) | (-0.049,0.213) |
| Private non-profit | -0.052 | -0.087 | -0.146^#^ | -0.057 | -0.048 |
|  | (-0.122,0.018) | (-0.212,0.037) | (-0.230,-0.062) | (-0.137,0.024) | (-0.120,0.023) |
| Private for-profit | -0.024 | 0.009 | -0.117^+^ | -0.012 | -0.030 |
|  | (-0.079,0.032) | (-0.057,0.075) | (-0.190,-0.044) | (-0.070,0.046) | (-0.091,0.030) |
| Beds | 0.000 | -0.002 | 0.014^#^ | -0.004 | -0.001 |
|  | (-0.011,0.011) | (-0.010,0.007) | (0.007,0.021) | (-0.011,0.003) | (-0.012,0.009) |
| Physicians | 0.001^#^ | 0.000 | -0.000 | 0.000^#^ | 0.001^+^ |
|  | (0.001,0.002) | (-0.000,0.001) | (-0.000,0.000) | (0.000,0.001) | (0.000,0.001) |
| Operating years | 0.001^+^ | 0.001^+^ | 0.000 | 0.001^*^ | 0.002^#^ |
|  | (0.000,0.003) | (0.000,0.002) | (-0.000,0.001) | (0.000,0.002) | (0.001,0.003) |

**Notes** Average marginal effects are reported; 95% confidence intervals (CIs) are in parentheses. All the regressions controlled for GDP per capita, urbanization rate, and population as well as a traditional Chinese medicine hospital dummy and a specialty hospital dummy. Number of beds were measured in units of 100 in the models. The standard errors were clustered at the county level.

**p* < .05, ^+^*p* < .01, and ^#^*p* < .001.
